# ST Quartz Acoustic Wave Sensors with Sectional Guiding Layers

**DOI:** 10.3390/s8074384

**Published:** 2008-07-25

**Authors:** Michael I. Newton, Paul Roach, Glen McHale

**Affiliations:** School of Science and Technology, Nottingham Trent University, Clifton Lane, Nottingham. NG11 8NS. United Kingdom

**Keywords:** ST-quartz, Love wave, guiding layer, SAW, SH-SAW

## Abstract

We report the effect of removing a section of guiding layer from the propagation paths of ST-quartz Love wave sensors; this offers the ease of fabrication of a polymer guiding layer whilst retaining the native surface of the quartz which may then be used for the attachment of a sensitizing layer. Data is presented for rigid and viscous loading, which indicates a small reduction in mass sensitivity compared to a Love wave device. Biosensing capabilities of these discontinuous ‘sectional’ guiding layer devices are demonstrated using protein adsorption from solution.

## 1. Introduction

Since the first reports of Love wave sensors on ST-quartz in 1992 [1, 2] there have been many investigations and applications of this system. Initial work used polymer guiding layers [1, 3, 4] with SiO_2_ later being used extensively. [5, 6] ST-quartz is known to support a Rayleigh wave parallel to the X-axis and a surface skimming bulk wave (SSBW) perpendicular to the X-axis which is launched at a shallow angle into the bulk [7]. The effect of this being an SSBW rather than a shear horizontal surface acoustic wave (SH-SAW) is known to have a contribution in addition to the Love wave guiding effect, with insertion loss of such devices initially reducing on the addition of an over layer [8-10]. This effect is thought to be due to the acoustic energy being confined closer to the substrate surface, which is not predicted by Love wave theory alone [9]. Further increasing the layer thickness beyond this minimum in insertion loss results in the attenuation of the signal, which is consistent with Love wave theory.

Although theoretical considerations for the Love wave model have been extensively investigated, there have been no reports that address the relative contribution of Love wave guiding layer and the SSBW effect on this technologically important cut of quartz. A practical motivation for such an investigation derives from the relative advantages of polymer and SiO_2_ guiding layers. Polymer guiding layers have the advantage of being simple to produce and can be incorporated as part of micro-fluidic structures [11, 12] whereas SiO_2_ guiding layers offer a more appealing surface for the attachment of a sensitizing layer for bio-sensing experiments but are much more difficult and expensive to fabricate [5]. To have the ease of fabrication of polymer guiding layers whilst retaining the native surface of the quartz for attachment of the sensitizing layer would represent a useful addition in the field of acoustic wave sensors.

Here we report the experimental investigation into the effect of removing a polymer guiding layer from a section of the propagation path whilst retaining it over the interdigital transducers (IDTs). Data are presented showing the enhanced sensitivity of so-called sectional guiding layer devices over an SSBW and comparing performance to a Love wave device having a complete guiding layer extending over both IDTs and the propagation path. Devices were fabricated on quartz substrates using an S1813 photoresist as a guiding layer. Mass sensitivities of devices having over-layers to cover the first mode were assessed. Gold deposition was used as a model for rigid mass loading whilst the effect of viscous loading was assessed using various water-glycerol mixtures. Bio-sensing capabilities of the sectional guiding layer devices were also demonstrated following protein adsorption from solution.

## 2. Experimental Section

**Figure 1.**
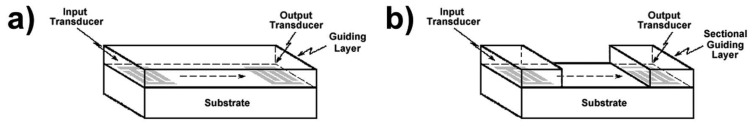
Schematic of a) Love wave devices and b) those having sectional guiding layers.

Acoustic wave devices were fabricated on ST-cut quartz with propagation orthogonal to the crystals X-direction. The IDTs consisted of sputtered gold (80 nm) using titanium (10 nm) as an adhesive layer, deposited using an Emitech K575X sputter coater. A double-double finger design was used with 6.75 μm finger widths and 4.5 μm spacings. IDTs were 40λ in length, with an aperture of 65λ and IDT centre-centre distance of 9 mm giving a fundamental frequency of 110 MHz. S1813 G2 (Shipley) was diluted in 2-ethoxyethyl acetate (Aldrich) being spun on the devices to form a guiding layer. Successive layers were built up baking to 130 °C between each coating. Layer thickness was verified using a Veeco Dektak 6M stylus profiler and an optical Veeco Dektak 3 surface analysis system. To compare devices having a complete guiding layer (Love wave) and a sectional guiding layer, the S1813 was removed completely from the propagation path (7 mm section) using a scalpel blade (Figure 1). Care was taken to ensure the substrate was not damaged. Successive 30 nm gold layers were deposited onto a defined area of 6 mm^2^ within the acoustic propagation path to assess frequency dependence of rigid mass loading, using an Emitech K575X.

Distilled water / glycerol (Sigma 98%) mixtures were likewise used to assess frequency dependence of viscous loading. Solutions were held over a defined area between the transducers using an o-ring seal and exchanged by flow, with measurements being taken under static conditions. Proteins adsorption experiments were conducted under continuous flow using Razel R-100EC syringe pumps set to deliver solutions at 1 mL min^-1^. Bovine serum albumin (Sigma, fraction V, lyophilized powder) solutions were prepared immediately prior to use at a concentration of 1 mg mL^-1^ in phosphate buffered saline (PBS, Sigma, pellets). Temperature stability of 24 ± 0.1 °C was achieved using an Octagon 10 incubator. An Agilent E5061A network analyzer was used to record frequency spectra. The acoustic devices were incorporated into an oscillator circuit as the feedback element for liquid studies. The circuit comprised amplifiers (Minicircuits ZFL-500LN), a 50 MHz high pass filter and 150 MHz low pass filter (Minicircuits BHP-50 and BPL-150), a directional coupler (Minicircuits ZFDC-10-2) and a frequency counter (Agilent 53132A) interfaced to a microcomputer.

## 3. Results and Discussion

**Figure 2.**
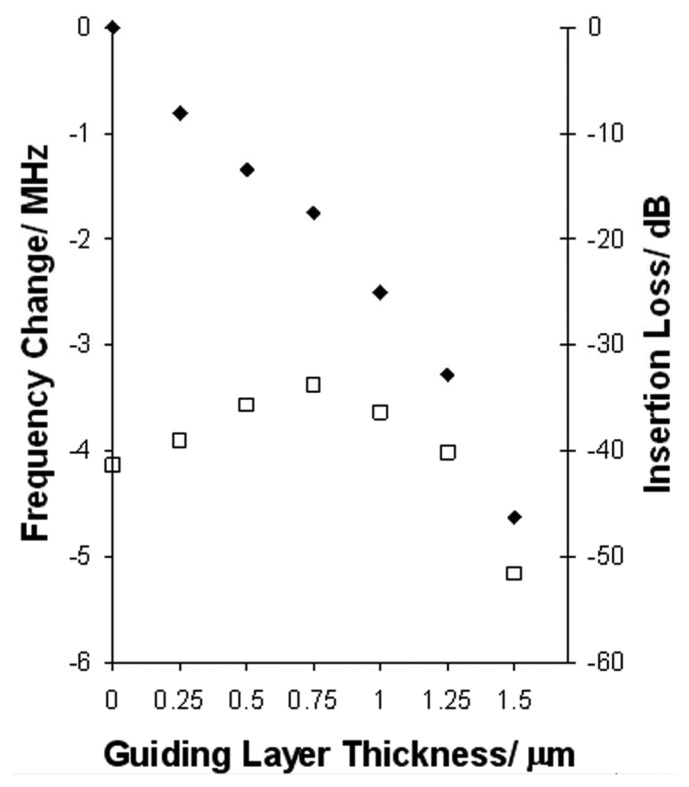
Insertion loss (open squares) and frequency change (solid diamonds) as a function of polymer guiding layer thickness showing the reduction in insertion loss up to 0.75 μm.

Guiding layers were initially constructed to cover the entire top surface of devices fabricated on quartz substrates using S1813 photoresist as guiding layer material. On bare quartz, an SSBW is launched at a shallow angle into the bulk. As the guiding layer thickness increases the wave travels closer to the surface of the substrate such that the device becomes more sensitive to surface perturbations. Figure 2 shows a decrease in frequency with increasing guiding layer thickness with an initial increase in signal strength up to a guiding layer thickness of 0.75 μm; this is the specific characteristic of the SSBW on ST-quartz that we exploit in this work. Further increase in guiding layer thickness results in signal attenuation due to mass loading which are consistent with previous reports.[9, 13] The choice of guiding layer thickness effectively sets the operating point for the use as a sensor.

Using gold to model rigid mass loading, the sensitivity of quartz devices was assessed for varying guiding layer thicknesses. Rigid mass loading of surface acoustic wave devices results in a proportional decrease in frequency; such a response is usually assumed to be due to a change in phase velocity allowing a Sauerbrey-type relationship to be applied [14]. For guided wave devices, a similar relationship holds, but with the overall sensitivity dependent on the operating point of the sensor on the dispersion curve [17]. The frequency change as a function of gold thickness was obtained for nine different guiding layer thicknesses up to 1.8 μm after which the guiding layer was removed from the propagation path and the gold deposition repeated. The maximum sensitivity for the continuous guiding layer was found to be 269 Hz ng^-1^ mm^-2^ and sectional guiding layer 195 Hz ng^-1^ mm^-2^ both corresponding to a thickness of 1 mm; further increase in guiding layer thickness resulted in a gradual decrease in sensitivity being comparable to those previously reported for similar systems [13].

**Figure 3.**
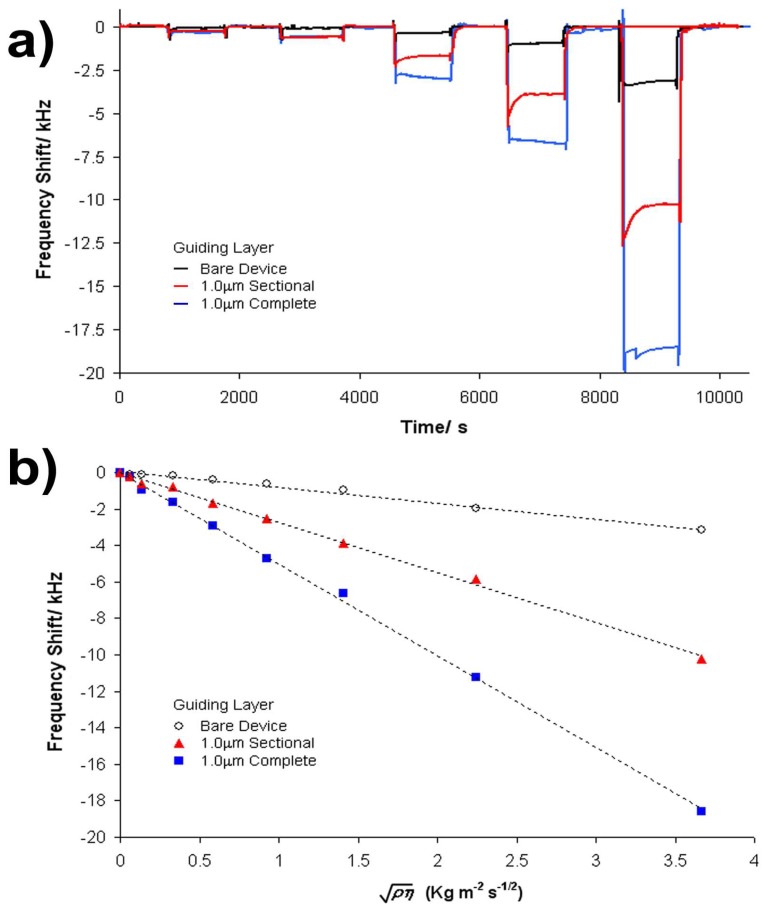
**(a)** Frequency response of black – bare device, red – 1 μm sectional guiding layer and blue – 1 μm Love wave device to varying glycerol-water mixtures exchanged over surface: 5, 10, 30, 50 and 70 % glycerol in distilled water. All data referenced to water. **(b)** frequency shift as a function of the square root of the density product for the bare device (-○-), 1 μm thick sectional guiding layer ( 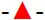) and 1μm thick continuous guiding layer ( 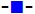).

The effects of viscous loading were investigated using the acoustic device as the feedback element in the oscillator circuit. The frequency was continually monitored whilst varying glycerol-water mixtures were exchanged over the sensor surface. The acoustic wave signal is attenuated by the liquid overlayer such that it decays with a penetration depth $\delta =\sqrt{\eta_{1}/(\pi f_{0}\rho_{l})}$, where *ρ_l_* and *η_l_* are the liquid density and viscosity respectively. The Kanazawa and Gordon relationship [18] between frequency change and square root of the density-viscosity product is assumed to hold for non-guided acoustic waves. Again, for guided wave devices a similar relationship holds, but with the overall sensitivity dependent on the operating point of the sensor on the dispersion curve [15]. Figure 3a shows examples of the flow profiles obtained, comparing the responses of an SSBW device (black line) to Love wave (blue line) and sectional guiding layer (red line) devices having a 1 μm thick guiding layer. Slight frequency disturbances were observed on the exchange of solutions, which are assumed to be due to local pressure fluctuations as the solutions are passed over the sensing surface. Measurements of frequency shifts relate to a water reference and the steady state glycerol-water mixture. Figure 3b shows the frequency change as a function of the square root of the viscosity density product comparing again an SSBW, Love wave and sectional guiding layer device having a 1 μm thick guiding layer. These data compare well to those previously reported for similar Love wave sensors [6, 13, 16].

It is clear that a quartz device launching a surface skimming bulk wave undergoes an enhancement of sensitivity if an overlayer is added, coving both the whole sensing surface and the IDTs or having a free propagation path. A main advantage to the latter design is that the sensing surface material is the quartz substrate. Previous studies have used Love wave sensors to follow bio(chemical)-surface interactions. It is often therefore necessary to metallize and chemically modify the sensing surface to adopt specific surface terminal chemistry, e.g. using gold-thiol self assembled monolayers (SAMs) [19-21]. By using a discontinuous guiding layer the benefits of enhanced sensitivity and ease of chemical modification are obtained. Moreover, if interaction with non-SAM surfaces are of interest, Love wave devices would be difficult to produce as additional material layers (such as SiO_2_ or quartz) would affect the operating point of the device. To demonstrate the use of sectional guiding layer devices for the study of (bio)molecule-surface interactions the adsorption of serum albumin onto bare quartz was followed.

**Figure 4.**
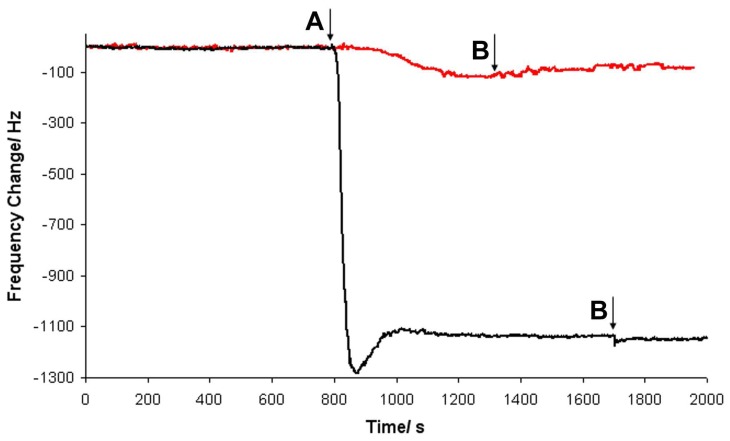
Albumin adsorption onto quartz followed by buffer rinse. Response of SSBW shown in red and sectional guiding layer device in black. Arrows indicate solution change: A) 1 mg mL^-1^ albumin solution and B) buffer rinse.

Figure 4 shows the flow profile observed for albumin adsorption onto the quartz substrate, comparing a SSBW device having no guiding layer and a sectional guiding layer device. Buffer was continually flowed over the sensing surface being exchanged for protein solution at time point A. The adsorption from solution is shown by a decrease in frequency. After the frequency became stable the solution was again switched to buffer to rinse any unbound protein from the surface. A much greater frequency shift was observed for the sensor having a guiding layer over the IDTs, demonstrating the ability of the sectional guiding layer devices for such applications.

## Conclusions

This work demonstrates a novel approach to producing acoustic wave sensors fabricated on ST quartz substrates having discontinuous, so-called ‘sectional’ guiding layers. Although the sectional guiding layer devices do not match the sensitivity of equivalent Love wave sensors, they have significantly higher sensitivity compared to a bare device. The presented device configuration has the advantage of presenting the substrate material as the sensing surface, allowing the convenient attachment of a sensitizing layer for bio-sensing experiments if required.
